# The Cheaper Cinchona Alkaloids

**Published:** 1875-09

**Authors:** 


The Cheaper Cinchona Alkaloids.—The last of the alkaloids—sulph. of cinchonidia—though *not the least*, comes up for our consideration. This article has not been so long upon the market as the latter alkaloid, in fact it has not till within a few months been brought more than casually to the notice of the profession; but because its introduction has been of but recent date, there is no reason why those who have seen fit to test its medicinal virtues and give it a fair and impartial trial should be treated so cavalierly when they attempt to call the attention of the profession to its value as a remedial agent. It is our object in this paper to call the attention, especially of country practitioners, to the unqualified value and merits of this drug as a tonic, febrifuge and antiperiodic. So far as our experience has gone with this alkaloid—cinchonidia sulph.—we are disposed to attribute to it very nearly, if not quite, an equivalent therapeutic value with that of the sulphate of quinine. We have used it in the same doses—ten to twenty grains—with complete success in interrupting the paroxysm of intermittents; we have administered it, in connection with morphia, to dispel the malarial complications that sometimes occur in pneumonia, with satisfactory results; and in acute rheumatism we have substituted it for quinia, whenever the latter drug seemed to be indicated, with unequivocal benefit. We have had this spring (1875) in this vicinity more than a usual amount of malarial or periodical diarrhœa and neuralgias, both among children and adults, for the relief of which we have been in the habit of prescribing the sulph. cinchonidia, with other appropriate remedies. In these cases the same amount was given as that of sulph. quinia under similar circumstances—we seldom or never had to repeat the dose—and its administration was attended with the most complete and satisfactory results.

This article—cinchonidia sulph.—can be obtained by the quantity for from seventy-five to eighty cents per ounce, and, therefore, we would respectfully ask the question: If the value of this agent as a tonic and febrifuge is equivalent, or nearly so, to that of quin. sulph., and its commercial value is only one-third the value of the latter, is it of no concern to the country practitioner, who has to furnish, at great expense, annually, his own drugs, not to the wealthy, but to the indigent from whom he never expects to receive one farthing for his services, and for whom he labors solely for the sake of suffering humanity, for the answer of a good conscience, and the gratitude of his beneficiaries?

We would not use these cheaper remedies merely from mercenary motives—no one could conscientiously do so; but we would and do prescribe and use them because we believe them, in equivalent doses, to be of the same therapeutic value as that of quinia, and that they may be safely used as a substitute for it; and in so doing we are treating our patients well, and, in not a few instances we are not only contributing our medicine and our services to the poor, but we are rendering good service to the general health and comfort of our patients, and saving to ourselves the difference of the cost of the drug used, which will amount to no mean sum in the course of the year, and which the country physician so much needs, because, at best his life is a hard one, and few there are, in deed, who make any more than enough, from year to year, for the economical support of themselves and families.—*Chicago Medical Journal.*

